# Health-Related Quality of Life in Adrenocortical Carcinoma: Development of the Disease-Specific Questionnaire ACC-QOL and Results from the PROFILES Registry

**DOI:** 10.3390/cancers14061366

**Published:** 2022-03-08

**Authors:** Rebecca V. Steenaard, Thomas M. A. Kerkhofs, Myrte Zijlstra, Floortje Mols, Michiel N. Kerstens, Henry J. L. M. Timmers, Rachel S. van Leeuwaarde, Koen M. A. Dreijerink, Elisabeth M. W. Eekhoff, Els J. M. Nieveen van Dijkum, Eleonora P. M. Corssmit, Ellen Kapiteijn, Marjolein N. T. Kremers, Richard A. Feelders, Harm R. Haak

**Affiliations:** 1 Department of Internal Medicine, Máxima MC, 5631 BM Eindhoven/Veldhoven, The Netherlands; h.haak@mmc.nl; 2 CAPHRI School for Public Health and Primary Care, Ageing and Long-Term Care, Maastricht University, 6229 HX Maastricht, The Netherlands; marjolein.kremers@mmc.nl; 3 Department of Research, Netherlands Comprehensive Cancer Organization (IKNL), 3511 DT Utrecht, The Netherlands; m.zijlstra@iknl.nl (M.Z.); f.mols@tilburguniversity.edu (F.M.); 4 Department of Internal Medicine, Division of Medical Oncology, Maastricht University Medical Center+, 6229 HX Maastricht, The Netherlands; thomas.kerkhofs@mumc.nl; 5 Department of Internal Medicine, St. Jans Gasthuis, 6001 BE Weert, The Netherlands; 6 Netherlands Association for Palliative Care (PZNL), 3511 DT Utrecht, The Netherlands; 7 Department of Medical and Clinical Psychology, Tilburg University, 5037 AB Tilburg, The Netherlands; 8 Department of Internal Medicine, University Medical Center Groningen, University of Groningen, 9713 GZ Groningen, The Netherlands; m.n.kerstens@umcg.nl; 9 Department of Internal Medicine, Radboud University Medical Center, 6525 GA Nijmegen, The Netherlands; henri.timmers@radboudumc.nl; 10 Department of Endocrine Oncology, University Medical Center Utrecht, 3584 CX Utrecht, The Netherlands; r.vanleeuwaarde@umcutrecht.nl; 11 Department of Internal Medicine, Amsterdam University Medical Centers, Location VUMC, 1081 HV Amsterdam, The Netherlands; k.dreijerink@amsterdamumc.nl (K.M.A.D.); emw.eekhoff@amsterdamumc.nl (E.M.W.E.); 12 Department of Surgery, Cancer Center Amsterdam, Amsterdam University Medical Centers, 1081 HV Amsterdam, The Netherlands; e.j.nieveenvandijkum@amsterdamumc.nl; 13 Department of Endocrinology, Leiden University Medical Center, 2333 ZA Leiden, The Netherlands; e.p.m.van_der_kleij-corssmit@lumc.nl; 14 Department of Medical Oncology, Leiden University Medical Center, 2333 ZA Leiden, The Netherlands; h.w.kapiteijn@lumc.nl; 15 Department of Endocrinology, Erasmus Medical Center, 3015 CA Rotterdam, The Netherlands; r.feelders@erasmusmc.nl; 16 Department of Internal Medicine, Division of General Internal Medicine, Maastricht University Medical Center+, 6229 HX Maastricht, The Netherlands

**Keywords:** adrenocortical carcinoma, health-related quality of life, questionnaire development, mitotane, chemotherapy

## Abstract

**Simple Summary:**

Patients with the rare cancer adrenocortical carcinoma are exposed to many symptoms and treatment side-effects. Research on how this can affect their health-related quality of life (HRQoL) is limited, however. This article includes the first assessment of HRQoL in a population-based cohort of patients with adrenocortical carcinoma with the European Organization for Research and Treatment of Cancer QLQ-C30 questionnaire and the newly developed disease-specific additional questionnaire ACC-QOL. The ACC-QOL has good psychometric properties in terms of validity, reliability, and responsiveness. Patients diagnosed more than 5 years ago reported a relatively good HRQoL compared with the Dutch reference population, but experienced residual fatigue and emotional problems. Patients after additional surgery reported a slightly lower HRQoL due to physical limitations. Patients who had recently received mitotane or chemotherapy reported a worse HRQoL and problems in many domains. This knowledge and the new disease-specific questionnaire can aid future research, side-effect monitoring, treatment guidance, and shared decision making.

**Abstract:**

We aimed to develop a disease-specific adrenocortical carcinoma (ACC) health-related quality of life (HRQoL) questionnaire (ACC-QOL) and assess HRQoL in a population-based cohort of patients with ACC. Development was in line with European Organization for Research and Treatment of Cancer (EORTC) guidelines, though not an EORTC product. In phase I and II, we identified 90 potential HRQoL issues using literature and focus groups, which were reduced to 39 by healthcare professionals. Pilot testing resulted in 28 questions, to be used alongside the EORTC QLQ-C30. In Phase III, 100 patients with ACC were asked to complete the questionnaires twice in the PROFILES registry (3-month interval, respondents: first 67, second 51). Confirmatory factor analysis demonstrated the structural validity of 26 questions with their scale structure (mitotane side-effects, hypercortisolism/hydrocortisone effects, emotional effects). Internal consistency and reliability were good (Cronbach’s alpha 0.897, Interclass correlation coefficient 0.860). Responsiveness analysis showed good discriminative ability (AUC 0.788). Patients diagnosed more than 5 years ago reported a good HRQoL compared with the Dutch reference population, but experienced residual fatigue and emotional problems. Patients who underwent recent treatment reported a lower HRQoL and problems in several domains. In conclusion, we developed an ACC-specific HRQoL questionnaire with good psychometric properties.

## 1. Introduction

Adrenocortical carcinoma (ACC) affects approximately one in a million persons each year [1]. When diagnosed at an early stage, surgery can result in long-term survival. However, often the prognosis is poor due to advanced disease or unfavorable tumor characteristics. Treatment options for ACC include surgery, mitotane therapy, chemotherapy and radiotherapy [2,3]. Each of these treatment options and the disease itself can have a negative impact on patients’ health-related quality of life (HRQoL).

HRQoL is usually assessed with questionnaires, which can be generic or for a specific disease group or issue. The EORTC QLQ-C30 is a well-known questionnaire designed for use among patients with cancer [4]. Its items include general effects of cancer on HRQoL such as general physical and emotional functioning, and symptoms such as fatigue. The EORTC also developed disease-specific questionnaires that can be used in addition to the EORTC QLQ-C30 questionnaire, for example, in breast cancer [5]. No disease-specific questionnaire has yet been developed for ACC and apart from questionnaires addressing hormone overproduction, there is no HRQoL questionnaire including items specific to ACC-related problems [6].

Even though HRQoL is an important part of cancer care, studies in ACC are limited [6]. To date, only the FIRMACT study has assessed HRQoL in a large study of patients with ACC [7]. They found a lower HRQoL as assessed with the EORTC QLQ-C30 in patients with advanced ACC compared to the general population. There are no publications yet that report specifically on HRQoL of patients with other stages of ACC or other treatment modalities.

Patients with ACC experience many combinations of issues which can impair HRQoL [8]. Examples of these issues are mitotane side-effects, hormone excess, problems with sexuality, and concerns about scan results. These issues are not yet included in the EORTC QLQ-C30 or any other questionnaire. The use of existing questionnaires in ACC research or patient care might consequently result in underreporting of HRQoL issues. We therefore believe that there is an unmet need for a disease-specific HRQoL questionnaire developed for use in patients with ACC.

This study aimed to develop and provide initial validation of a disease-specific HRQoL questionnaire to be used alongside the EORTC QLQ-C30 in patients with ACC (ACC-QOL). The secondary objective is to assess HRQoL in a population-based cohort of ACC patients.

## 2. Materials and Methods

The method followed the guidelines published by the EORTC QOL Group for phases I to III of new questionnaire development [5]. However, since the study was not initiated by an EORTC working group, and the development of this questionnaire was not set up as an international study, the questionnaire cannot be seen as an official EORTC product. The study has been approved by the Dutch-certified Máxima MC Committee for Ethics (approval number N19.075).

### 2.1. Phase I—Issue Identification

In the first phase, a comprehensive list was composed of potential HRQoL issues that patients with ACC might face. This was done using a systematic literature search and focus group interviews, of which the methods and results can be found in more detail in previous publications [6,8]. In short, we found 6 studies that assessed HRQoL in patients with ACC and listed the reported issues. We then organized focus group interviews with 10 patients and their partners purposefully selected to form a heterogeneous group (mean age 57.1 years, range 26–71; 7 females; mean time since diagnosis 4.9 years, range 1–18; 2 cortisol production, 1 cortisol and androgens production; 4 ENSAT stage I-II, 1 stage III, 5 stage IV; 10 surgery, 6 mitotane, 2 radiotherapy; 5 complete response, 3 stable disease, 2 progressive disease).

We developed a semi-structured interview guide based on the literature search and clinical expertise of the research group [6]. Patients were asked to describe their experiences with the disease and treatment options, the impact on everyday life, and their concerns regarding HRQoL. The interviews were transcribed smooth verbatim and transcripts were analyzed by inductive qualitative content analysis using QDA miner lite software v2.0.5 [9,10,11]. Coding was performed by two independent researchers and discrepancies were resolved through discussion. The codes and the results from the literature search were used to form a comprehensive list of 90 HRQoL issues.

### 2.2. Phase II—Question Selection

Five healthcare professionals (HCP) were selected based on their experience in treating patients with ACC. The group consisted of two endocrinologists, two oncologists and one surgeon employed at adrenal expert centers in the Netherlands. The HCPs evaluated the list of HRQoL issues produced in phase I and were asked to identify missing issues. They were then asked to rate issues (excluding issues already in the EORTC QLQ-C30) based on relevance using a four-point Likert scale (not relevant–very relevant) and select 10 core issues to be included in a disease-specific HRQoL questionnaire. The most relevant items on the list were selected based on the Likert scale rated by the HCPs (mean score ≥ 2.5) and the core issues selected by the HCPs (selected by ≥1 HCP). This resulted in a list of 39 relevant HRQoL issues.

The list of relevant HRQoL issues was transformed into questions compatible with the EORTC QLQ-C30 format (response categories: “not at all”, “a little”, “quite a bit” and “very much”; time frame: one week for all items, 4 weeks for the sexuality item). Issues already used in other EORTC models, were selected from the EORTC QOL Item Bank, which comprises all existing EORTC QOL questionnaire items and their translations [12]. The issues with no corresponding question in the EORTC item bank (*n* = 13) were converted into questions using the same format after consensus by two authors.

The questions were pilot tested using cognitive interviews with four consecutive patients with ACC at the Máxima MC outpatient clinic. They were asked to complete the questionnaire using the thinking-out-loud method and then answer questions regarding readability, feasibility, relevance, and identification of repeated themes. This resulted in a further reduction of the questionnaire to 28 questions. The four participants needed between 15 and 30 min to complete both the EORTC QLQ-C30 and the ACC-QOL.

### 2.3. Phase III—Validation

Questionnaires were collected within the Patient Reported Outcomes Following Initial treatment and Long term Evaluation of Survivorship (PROFILES) registry [13]. PROFILES is a registry for the study of the physical and psychosocial impact of cancer and its treatment from a dynamic, growing population-based cohort of both short and long-term cancer survivors. The registry allows patients to complete questionnaires online or on paper. Patients for the questionnaire validation phase were selected from the Netherlands Cancer Registry. This registry includes data on all cancer patients diagnosed in the Netherlands. Inclusion criteria were diagnosis with ACC, age above 18 years at diagnosis, alive in March 2021, and treated in an adrenal expert center in the Netherlands. Principal investigators from each adrenal expert center checked the selection (130 patients) and excluded patients who would not be able to fill in a questionnaire (e.g., language barrier, impaired cognitive status, deceased between selection and questionnaire administration).

The principal investigators sent an invitation letter to the remaining 100 patients with a link to the online informed consent form and the EORTC QLQ-C30 and ACC-QOL questionnaires. Paper informed consent forms and questionnaires were sent upon request. When a patient did not respond within 4 weeks, a reminder was sent. The second questionnaire was sent to the respondents 3 months later and contained a global rating scale assessing change in overall HRQoL to assess responsiveness (how is your quality of life compared to 3 months ago? Response categories: “worse”, “stable”, “better”).

EORTC QLQ-C30 sum scores were calculated using the equations provided by EORTC [14]. The EORTC QLQ-C30 overall score, global health score and function scores, including physical, role, emotional, cognitive, and social functioning, were scaled from 0 to 100 where 100 equals perfect HRQoL or function. The symptom scores, including fatigue, nausea and vomiting, pain, dyspnea, insomnia, appetite loss, constipation, diarrhea, and financial difficulties, were scaled from 0 to 100, where 0 equals no symptoms. Clinical data of the respondents, the non-respondents, and excluded patients were collected from the Netherlands Cancer Registry to assess selection bias and to determine the effect of clinical characteristics on HRQoL.

### 2.4. Data Analysis

We used descriptive statistics for Phase I-III to determine item relevance. Differences in patient characteristics between respondents, non-respondents, and excluded patients were determined using ANOVA tests for continuous variables and chi-square (or Fisher’s exact) tests for categorical variables. Analyses were performed in SPSS version 26 when unspecified or in STATA version 17 when specified.

Psychometric properties of the ACC-QOL were determined according to the COSMIN taxonomy and methodology, by means of the classical test theory [15,16]. Face validity was determined in phases I and II of the study. In phase III, construct validity was assessed using confirmatory factor analysis to confirm the hypothesized scale structure. Four models with increasing number of scales were tested by standard equation modelling in STATA version 17. Model 1: no scale structure; model 2: physical effects and emotional effects scales; model 3: mitotane side-effects, hypercortisolism/hydrocortisone effects and emotional effects scales; model 4: food, neurology, skin, hypercortisolism/hydrocortisone effects and emotional effects scales. Items with low correlation (r < 0.2) to any scales and the overall score were considered for deletion. We hypothesized that either model 3 or 4 would have the best fit. A comparative fit index (CFI) close to 0.95 and a standardized root mean square residual (SRMR) and root mean square error of approximation (RMSEA) close to 0.08 was considered a good-fitting model. The best-fitted model was used to calculate the scales scores and overall score for the ACC-QOL in the same manner as the EORTC QLQ-C30 symptom and overall scores [14]. Construct validity was further confirmed by hypothesis testing. A hypothesis was confirmed if a correlation was below <0.4 for scales in the ACC-QOL without relations to scales in the EORTC QLQ-C30, and between 0.4 and 0.9 for scales with a relation. Negative correlations in the same magnitude were considered for scales with opposite direction. Any correlation above 0.9 would be rejected, because a scale would not have an added value to the scale in the EORTC QLQ-C30. Because the scale mitotane side-effects was hypothesized to have many correlations with the EORTC QLQ-C30 subscales, the hypotheses were formed for the scales food, neurology, and skin, respectively, irrespective of the best-fitted model. Validity was determined to be acceptable if 7 out of 10 hypotheses were confirmed.

Reliability of the overall ACC-QOL score was assessed using the interclass correlation coefficient (ICC) for absolute agreement between the overall ACC-QOL scores of the first and second questionnaire in patients who perceived a stable HRQoL. An ICC of >0.7 was considered good. Measurement error of the overall ACC-QOL score was calculated as the standard error of the mean (SEM) for the difference between overall ACC-QOL scores of the first and second questionnaire in patients who perceived a stable HRQoL. Reliability of the individual items was calculated with linear weighted Cohen’s Kappa for agreement in the response options between the two assessments of patients who perceived a stable HRQoL (STATA version 17). Interpretation of the Kappa value was performed according to the definition of Landis and Koch [17]. Internal consistency was determined by the Cronbach’s alpha of the overall score and the separate scales. A Cronbach’s alpha of >0.7 was considered good.

Responsiveness was assessed with the criterion approach using a global rating scale for perceived HRQoL change (“worse”, “stable”, ”better”). The change in overall ACC-QOL score was calculated for each patient who participated in both the first and second questionnaire. The mean change was compared between the categories of improvement by means of an ANOVA analysis and by Spearman’s correlation. A Spearman’s rho above 0.4 was considered good. A ROC-analysis was used to determine the ability of the change in overall ACC-QOL score to discriminate between patients with deterioration and patients with stable or improved HRQoL. An area under the curve (AUC) above 0.7 was considered good.

To determine the effect of disease stage and treatment options on HRQoL, difference in EORTC QLQ-C30 and ACC-QOL scores between different patient groups were calculated using ANOVA tests.

## 3. Results

The first questionnaire was completed by 67 patients, the second by 51 patients. We found no differences between respondents, non-respondents, and excluded patients, except for a high proportion of mitotane users among the respondents (Table 1). The majority of the patients were long-term survivors (>5 years since diagnosis, *n* = 32) with a median time since diagnosis of 6 years (range 0–38 years). A total of 20 patients had received active treatment within the last 1000 days, including surgery, adjuvant radiotherapy on the tumor bed, mitotane therapy or chemotherapy. The mean age at the time of the questionnaire was 59 years (range 29–83 years).

### 3.1. Psychometric Properties of ACC-QOL

The distribution of the response options for each item in the ACC-QOL is shown in Table S1. Each item had a low number of missing values (0–3.9%).

Construct validity was assessed with confirmatory factor analysis using four different models with an increasing number of scales (Table 2). As hypothesized, model 3 with three scales (mitotane side-effects, nine items, hypercortisolism/hydrocortisone effects, four items, emotional effects, ten items) and three items (physical limitations due to surgery, virilization and need for peer support) had the best fit. However, the CFI was considered moderate (0.667). The items on weight gain and social support had limited correlation with any of the scales and with the overall score (weight gain-overall r 0.08; social support-overall r 0.138) and were therefore deleted from the model. Model 3 was used to calculate the overall score for the ACC-QOL. The construct validity was further confirmed by hypothesis testing. Eight out of ten hypotheses about the relations between the ACC-QOL scales and EORTC QLQ-C30 scales were confirmed, and no correlations above 0.9 were seen (Table 3).

Reliability of the overall ACC-QOL score was good, with an ICC for absolute agreement of 0.860 in the patients who perceived a stable HRQoL. The measurement error of the overall ACC-QOL in stable patients was low (mean difference ±SEM: −1.03 ± 1.27). Reliability of the individual items in the score was fair for two items and moderate to substantial for 24 out of 26 items in patients with perceived stable HRQoL (weighted Cohen’s Kappa 0.34–0.86). The overall ACC-QOL score and the three scales had a good internal consistency (Cronbach’s alpha overall 0.897, mitotane side-effects 0.763, hypercortisolism/hydrocortisone effects 0.784, emotional effects 0.875).

Responsiveness was assessed using a global rating scale for perceived HRQoL change (“worse” *n* = 11, “stable” *n* = 29, “better” *n* = 10, missing *n* = 1). There was a gradual increase in overall ACC-QOL change score for the categories of improvement (mean change ±SD: “worse” −6.27 ± 6.22, “stable” −1.03 ± 6.82, “better” 5.38 ± 5.45; ANOVA *p* < 0.001). The correlation between the change scores and the categories of improvement was good (Spearman’s rho 0.548). ROC analysis showed the change in overall ACC-QOL score had a good ability to discriminate between patients with deterioration (*n* = 11) and patients with stable or improved HRQoL (*n* = 39), with an AUC of 0.788.

### 3.2. HRQoL Scores in Patient Groups

**Figure 1:**
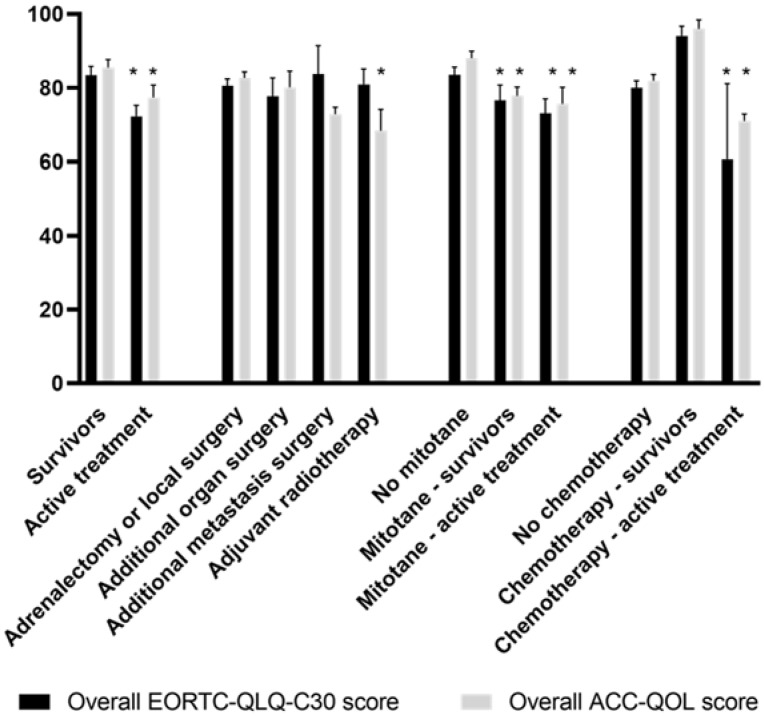
Mean overall EORTC QLQ-C30 and ACC-QOL scores for different patient groups. Error bars represent standard error of the mean. * *p* < 0.05.

The mean overall EORTC QLC-C30 score for the total population was 80.5 (Table S2). We found no differences in HRQoL between males and females or between patients with different ENSAT stages at diagnosis. The overall score was significantly lower for patients who underwent active treatment within the last 1000 days, including surgery, adjuvant radiotherapy, mitotane therapy or chemotherapy (mean overall EORTC QLQ-C30 score 72.2) (Figure 1). These patient groups reported a significantly lower HRQoL in several domains, including lower role, emotional and cognitive functioning, and a higher fatigue score on the EORTC QLQ-C30 and higher mitotane and emotional scores on the ACC-QOL.

Patients who underwent additional surgery (e.g., nephrectomy) or metastatic surgery (e.g., liver or lung resection) reported more physical limitations due to surgery on the ACC-QOL than patients who were only treated with an adrenalectomy or local surgery. Patients who were treated with radiotherapy on the primary tumor bed after surgery reported similar physical limitations due to surgery as patients after metastatic surgery. They had the lowest overall ACC-QOL score, with a significantly higher hypercortisolism/hydrocortisone score.

Patients who had received mitotane therapy within the last 1000 days, had significantly lower overall EORTC QLQ-C30 and ACC-QOL scores compared to those never treated with mitotane. These patients indicated problems in many domains, including role, emotional and social functioning and in fatigue, dyspnea, mitotane, emotional and peer support. Patients who had received mitotane therapy at any timepoint during the treatment period, even longer ago, also reported significantly lower scores in these domains compared to those never treated with mitotane. The two patients who had received chemotherapy within the last 1000 days, scored significantly lower on both questionnaires. They had significantly worse scores in physical functioning, fatigue, nausea, constipation on the EORTC QLQ-C30 and the emotional score of the ACC-QOL. The five patients that had received chemotherapy longer ago had similar HRQoL scores to the ACC survivors.

Patients who were diagnosed more than 5 years ago had a relatively good overall EORTC QLQ-C30 score (mean 83.4). However, they scored relatively high on symptom scores fatigue (mean 29.8) and insomnia (mean 23.8) from the EORTC QLQ-C30 and the emotional score (mean 22.6) from the ACC-QOL.

When looking at the individual items from the ACC-QOL, two items had a high percentage of respondents who answered “quite a lot” or “very much”. These items decreased physical capability experienced because of the disease (49.3%), and a negative effect of the disease on sex life (38.8%). Furthermore, the fatigue and emotional scores were worse in all patient groups. This indicates that the majority of ACC patients experience fatigue and emotional problems, which can persist for a long time after diagnosis and the completion of therapy.

## 4. Discussion

We presented the first disease-specific HRQoL questionnaire to be used in patients with ACC, the ACC-QOL. This questionnaire can be used alongside the EORTC QLQ-C30 to obtain a complete picture of the HRQoL of a patient with ACC. The proposed version of the ACC-QOL consists of 26 questions and includes 3 scales (mitotane side-effects, hypercortisolism/hydrocortisone effects and emotional effects) and three single items (physical limitations due to surgery, virilization, need for peer support). The questionnaire has good psychometric properties in terms of face and construct validity, reliability, and responsiveness.

HRQoL assessments are important for both research and clinical care of patients with ACC. In trials, HRQoL is often used as a secondary outcome. A questionnaire should be sensitive to all HRQoL issues that can be caused by the disease and treatment under study. The currently available HRQoL questionnaires did not include effects of mitotane side-effects, hormone production or hydrocortisone substitution and might therefore lack sensitivity to detect all HRQoL issues relevant for patients with ACC. HRQoL questionnaires can also be used to monitor side-effects during treatment, to improve patient–doctor communication, and to aid in treatment decisions [18,19]. A disease-specific questionnaire is useful for detecting all issues relevant for the patient. The ACC-QOL is designed to be used alongside the EORTC QLQ-C30 and together the two questionnaires provide a more precise evaluation of HRQoL in patients with ACC, especially during active treatment. It can therefore be used in research setting and patient follow-up, without the added use of a general HRQoL questionnaire to reduce patient burden.

Besides the development and initial validation of the first disease-specific HRQoL questionnaire for patients with ACC, this study is also the first to assess HRQoL in a population-based cohort of ACC patients. We discovered that the overall HRQoL of ACC survivors is good, but slightly lower compared to other cancer survivors in a Dutch reference population (83.4 versus 88.3, respectively) [20]. Despite the relatively good overall score, ACC survivors and patients during all treatment modalities score high on fatigue and emotional problems. These patients might benefit from early referral to rehabilitation or psychology services for extra support [21]. Patients who had received recent active treatment, mostly mitotane therapy and chemotherapy, experienced additional issues in many HRQoL domains. In the current study we cannot discriminate between complaints due to tumor progression and actual treatment side-effects. The data, however, indicate that patients with recent active treatment experience many issues that influence their HRQoL. Using a HRQoL questionnaire, and especially a disease-specific questionnaire such as the ACC-QOL, can help identify these issues during treatment and follow-up. This can lead to better patient–doctor communication, treatment guidance and shared decision making [22].

The current study has some potential limitations. The number of patients in this study is low due to the rarity of ACC. Every patient ever diagnosed with ACC in the Netherlands who wase eligible for the study was invited to participate. The number of included patients was sufficient for the planned analyses, according to the methodology of classical test theory [15]. The questionnaire developed in this study needs further international validation following the phase IV methodology, in agreement with the EORTC guidelines [5]. Another limitation is the potential survivor bias [23]. The population included a relatively high number of patients who were free from disease after treatment, which accounts for the high mean overall HRQoL scores. However, one-third of the population had recent treatment and the findings for the total population are comparable to HRQoL found in other cancer survivors [20]. In addition, no difference was found in the patient characteristics between respondents, non-respondents and excluded patients, which indicates the results can be extrapolated to the entire Dutch population of patients with ACC.

## 5. Conclusions

We generated the first disease-specific questionnaire for assessing HRQoL in patients with ACC with good psychometric properties, to be used in addition to the EORTC QLQ-C30 questionnaire. We found that ACC survivors reported a good HRQoL with residual fatigue and emotional problems. Patients who underwent recent treatment for ACC reported a lower HRQoL and experienced problems in many domains.

## Figures and Tables

**Table 1:** Characteristics of respondents, non-respondents, and excluded patients.

	Respondents *n* = 67	Non-Respondents *n* = 33	Excluded Patients *n* = 30	*p*-Value
Age at diagnosis (mean years)	50.9	51.2	48.4	0.75
Female	38	24	22	0.15
ENSAT stage				0.16
I	8	2	1	
II	24	12	5	
III	10	6	5	
IV	6	4	2	
Treatment modality				
Adrenalectomy	59	28	27	0.66
Local surgery	6	4	4	0.81
Additional or metastasis surgery	11	2	4	0.33
Radiotherapy	5	2	0	0.66
Mitotane	28	6	6	0.02
Chemotherapy	5	3	2	0.93

**Table 2:** Confirmatory factor analysis.

	CFI > 0.95	RMSEA < 0.08	SRMR < 0.08
Model 1: Overall	0.527	0.139	0.124
Model 2: Physical effects + emotional effects + overall	0.613	0.127	0.126
Model 3: Mitotane side-effects + hypercortisolism/hydrocortisone effects + emotional effects + overall	0.667	0.117	0.116
Model 4: Food + neurology + skin + hypercortisolism/hydrocortisone effects + emotional effects + overall	0.511	0.139	0.138

CFI: comparative fit index; RMSEA: root mean square error of approximation (RMSEA); SRMR: standardized root mean square residual.

**Table 3:** Hypothesis testing of scale structure correlations between ACC-QOL scales and EORTC QLQ-C30 subscales.

ACC-QOL Scale	EORTC QLQ-C30 Subscale	Hypothesis Confirmed If	r	Confirmed?
Overall ACC-QOL	Overall EORTC QLQ-C30	r 0.40–0.90	0.657	Yes
Mitotane-food	Appetite	r 0.40–0.90	0.409	Yes
Mitotane-neurology	Cognitive function	r −0.40–−0.90	−0.523	Yes
Mitotane-skin	None	r < 0.40	<−0.403	No
Hypercortisolism/ hydrocortisone	Emotional functioning	r −0.40–−0.90	−0.462	Yes
Emotional	Emotional functioning	r −0.40–−0.90	−0.613	Yes
Emotional	Social functioning	r −0.40–−0.90	−0.661	Yes
Operation	Physical functioning	r −0.40–−0.90	−0.397	No
Virilization	None	r < 0.40	<0.260	Yes
Peer support	None	r < 0.40	<0.309	Yes

## Data Availability

The data presented in this study are available in this article.

## Supplementary Materials

The following supporting information can be downloaded at: https://www.mdpi.com/article/10.3390/cancers14061366/s1, Table S1: Distribution of response options per ACC-QOL item; Table S2: Group differences in mean EORTC-QLQ-C30 and ACC-QOL scores.
